# Biomathematical model to analyze the transmission dynamics of Covid-19: Case study, Santiago de Cali, Colombia

**DOI:** 10.1371/journal.pone.0311414

**Published:** 2024-12-02

**Authors:** Miguel Alfaro, Carlos Rubio, Guillermo Fuertes, Manuel Vargas, Armando Mejia-Giraldo

**Affiliations:** 1 Industrial Engineering Department, University of Santiago de Chile, Santiago, Chile; 2 Facultad de Ingeniería, Universidad de San Buenaventura, Cali, Colombia; 3 Facultad de Ingeniería, Ciencia y Tecnología, Universidad Bernardo O’Higgins, Santiago, Chile; Norbert Wiener University, PERU

## Abstract

The Covid-19 pandemic has challenged both the scientific community and government authorities in Colombia. Both sectors are collaborating to understand the transmission and spread of the virus and to establish control strategies. This study proposes a biomathematical model with difference equations to analyze the transmission of Covid-19 in Santiago de Cali from March 2020 to June 2022. The results indicate that most of the inhabitants could be positive at some point, but with containment measures, a manageable number of symptomatic cases could be maintained. In addition, the cumulative fatality curve is fitted to the Gompertz model. The method used for parameter fitting or estimation was Gauss-Newton. This approach provides valuable information for decision making and pandemic management in the city.

## Introduction

Severe acute respiratory syndrome coronavirus 2 (SARS-CoV-2) is a virus of the coronaviruses family that causes Covid-19 respiratory infection. This virus has a zoonotic origin, i.e., it was transmitted from an animal host to a human host and subsequently from human to human [1]. The bat is the natural host of SARS-CoV-2, and its mechanism of transmission to humans is through respiratory secretions and/or material from the animal’s digestive tract [2]. Among humans, transmission of the disease occurs both directly and indirectly through respiratory tract fluids [3].

The World Health Organization (WHO) has stated that approximately one in five people infected with Covid-19, i.e., 20%, develop severe disease with respiratory distress [4]. In addition, Berlin et al. [5] warns of higher risks of complications in older patients and in those with respiratory, glycemic, cardiac, oncologic or blood pressure diseases.

The infection was reported on November 17 in Wuhan, capital of Hubei, China. However, WHO notes that public health entities in the Republic of China report that the first proven case occurred on December 8, 2019, in Wuhan [2]. The global spread of SARS-CoV-2 and the thousands of deaths caused by Covid-19 led WHO to declare a pandemic on March 12, 2020 [6]. Then, in the city of Santiago de Cali, the National Institute of Health (NIH) [7] of Colombia confirmed the first positive case on March 13, 2020. This was done using the rapid and accurate SARS-CoV-2 detection technique enabled by real-time reverse transcription-polymerase chain reaction (RT-PCR) [8]. RT-PCR can detect SARS-CoV-2 nucleic acids present in nasopharyngeal fluids [9].

For Lee and Thacker [10] a fundamental concept in clinical epidemiology is Public Health Surveillance (PHS) systems, defined by WHO as the systematic practice of collecting, analyzing, interpreting and disseminating health data for the planning, implementation and evaluation of public health actions [4]. Since the beginning of the Covid-19 pandemic, PHS in Colombia [11], together with academic researchers, has focused its efforts on paying special attention to epidemiological surveillance. For Alfaro et al. [12] the interest has been focused on the one hand, on clarifying the true trends of the data series of infected, deceased and recovered cases and, on the other hand, on designing and implementing public policies and health measures that have a significant and differential impact on the control of the epidemic. According to Rubio et al. [13], modeling and simulation are fundamental tools for decision-making. They can be used to develop epidemiological surveillance strategies, which are important for controlling the spread of diseases [14]. However, it is important to keep in mind that each disease has its own biological characteristics, so models must be individually adjusted to address specific situations [15].

Different authors have adapted and simulated mathematical models for the purpose of understanding Covid-19 propagation processes and facilitating decision making. Zhang et al. [16] present a detailed study on the evolution of the epidemiology and transmission dynamics of Covid-19 outside Hubei, China, focusing on geographical analysis, transmission factors, and control measures. Garba et al. [17] developed a model to analyze the transmission dynamics of the Covid-19 pandemic in South Africa. As a result, they were able to identify transmission patterns, estimate the burden of disease and evaluate interventions to control the spread of the virus. Similarly, Fang et al. [18] analyzed the dynamics of Covid-19 spread and evaluated the effectiveness of government interventions using data. As a result, they identified trends in virus transmission and assessed the impact of measures implemented by the government in containing the outbreak. Agosto and Giudici [19] propose an autoregressive Poisson model to understand the transmission dynamics of Covid-19, achieving a more accurate estimate of the transmission rate based on historical data. Also, Xiang et al. [20] presented a methodology based on the TVP-VAR model to model the global and dynamic spread of Covid-19. Dynamic contagion indices are constructed from two perspectives, (i) pairwise net dynamic connectivity and (ii) directional dynamic connectivity. The results allow us to understand the spread of the virus and make informed decisions for pandemic control.

Recently, Gayawan et al. [21] using different statistical techniques, including a two-parameter obstacle Poisson model, analyzed the spatiotemporal dynamics of the Covid-19 epidemic in Africa. They were able to identify patterns of virus spreading in different regions of the continent and to understand how the pandemic has evolved over time in this area. In addition, Albertin et al. [22] evaluated the effect of different ventilation strategies to mitigate the risk of Covid-19 infection in educational buildings. The methodology of this study proposes using MatLab software, a mass balance of CO_2_ and Covid-19 particles and a Monte Carlo simulation to iteratively evaluate the risk of contagion. The results show some ventilation strategies to reduce the risk of contagion. Similarly, Roques et al. [23] evaluated the impact of confinement on the epidemic dynamics of Covid-19 in France. These authors proposed the use of a mechanistic model for the description of the epidemiological process, a probabilistic observation model and an inference procedure. The study shows that confinement reduced the number of effective reproductions. The importance of maintaining a low value of effective reproduction to prevent a second uncontrolled wave is emphasized. Samui et al [24] propose a mathematical model to describe the transmission dynamics of Covid-19 in India. The model considers factors such as infection rate, basic reproduction number, and interventions implemented. The model simulations, through different scenarios, capture the increasing trend of the course of the Covid-19 epidemic. Singh et al. [25] developed a compartmental mathematical model to study the transmission dynamics of Covid-19 and tuberculosis. The model shows that Covid-19 can increase tuberculosis transmission, especially in populations with high levels of tuberculosis. In addition, Pelinovsky et al. [26] present a novel mathematical model based on the Gompertz equation to simulate the spread of Covid-19, fitted to pandemic data in China. It predicts with reasonable accuracy the number of cases and reveals influential factors in the spread of the virus.

### Contributions and limitations

This study analyzes the evolution of the Covid-19 pandemic in the city of Santiago de Cali through three dimensions. (1) descriptive dimension: in this dimension, the behavior of the data series related to the pandemic is examined; (2) deterministic dimension: the dynamic system is modeled using a set of equations in autonomous differences; and (3) inertial dimension: the calibration of the Gompertz model is carried out in relation to the accumulated death data.

A mathematically modeled scenario is presented for a particular period. The aspects of epidemiological equilibrium and stability in relation to the category of the deceased population are obtained by fitting the observed dynamics with the Gompertz curve.

The psychosocial dimension is examined in relation to health entity guidelines and local incidence and mortality data. This is done at the local level, considering the context of the social outbursts that took place in the city.

This paper focuses on the study of time series of susceptible, infected, recovered and deceased from a deterministic perspective using a system of difference equations. Therefore, no cross-sectional data or geospatial techniques were used in this research. In addition, confinement constraints precluded field data collection, so only data provided by the NIH was used.

The remainder of this paper is organized as follows: section 2 outlines the methodology used in the study, providing a detailed description of each of the steps that make up the model. Section 3 presents the results of the case study. Finally, section 4 presents the conclusions of the research.

## Methodology and data

### Research design

To characterize the dynamic spread of Covid-19 in Santiago de Cali, a discrete-time biomathematical simulation model, supported by difference equations, was used. To introduce the use of deterministic tools of mathematical epidemiology, aspects such as (1) epidemiological subdivision of a population; (2) effective contact rate; (3) competing risks; (4) transition rate; and (5) basic reproductive number are described. The psychosocial dimension of social indiscipline in outbreaks of protests and other phenomena such as population agglomerations in sports venues is analyzed in contrast with the recommendations of health entities and the incidence and mortality figures by Covid-19. This document highlights the importance of the medical-epidemiological aspects, which provide information on the dynamics of the disease, and of the mathematical-epidemiological aspects, which help to model the transmission dynamics. Both contribute to ensuring the relevance and effectiveness of prevention policies.

### Methodology

S1 Table shows observed data on infected, recovered and deaths in the city of Santiago de Cali, from March 13, 2020 to June 30, 2022. For short periods, fluctuations in the growth rates and level of infected can be explained by the agility in taking samples for the detection of new cases and obtaining timely results. For long periods, the trends observed are due to structural circumstances such as public policies to contain the epidemic, epidemiological surveillance, and attention to the health sector. Based on the information in S1 Table.

**Fig 1:**
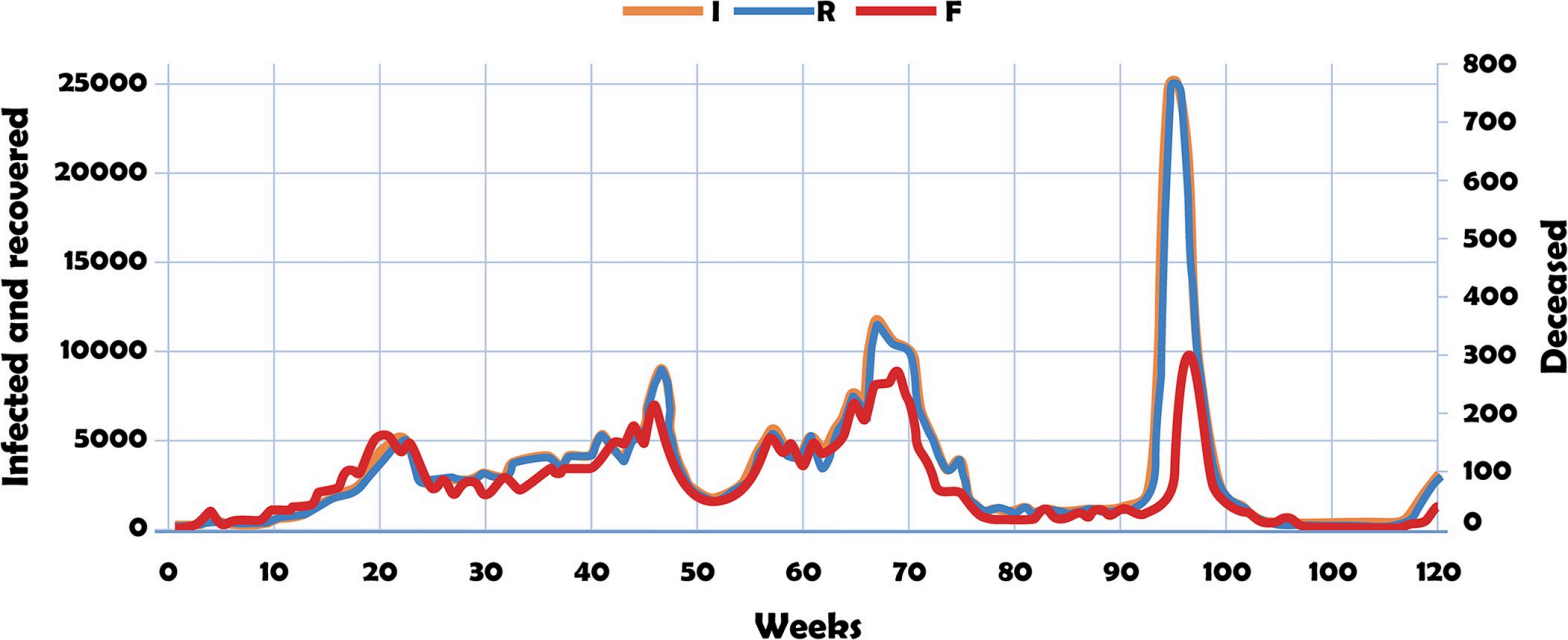
Dynamics of infected (I), recovered (R) and deceased (F). Data observed in Santiago de Cali city: 13/03/2020–30/06/2023.

Fig 1 shows the dynamics of infected, recovered and deceased, data observed in Santiago de Cali in the period 13/03/2020–30/06/2022.

Table 1 presents a summary of the statistics of infected, recovered and deceased persons during the first four waves of Covid-19 in Santiago de Cali, organized by duration in weeks.

**Table 1:** Observed data for the first four waves of Covid-19.

Surge	Duration	Statistic	I	R	F
1	27	Total	44.240	42.347	1.615
		Mean: $\bar{x}$	1.639	1.568	60
		Sta.Dev.: *σ*	1.540	1.482	51
2	21	Total	89.685	86.988	2.230
		Mean: $\bar{x}$	4.271	4.142	106
		Sta.Dev.: *σ*	1.642	1.598	44
3	28	Total	136.759	132.823	3.297
		Mean: $\bar{x}$	4.884	4.744	118
		Sta.Dev.: *σ*	3.086	3.002	78
4	14	Total	93.235	91.457	1.053
		Mean: $\bar{x}$	6.660	6.533	75
		Sta.Dev.: *σ*	8.677	8.573	94

### Elements of mathematical epidemiology for Covid-19

Kermack and McKendrick [27] established the basis for what is known as the threshold effect, stating that there is a minimum value N of individuals in a population, so that, if the number of individuals is greater than this minimum, then there is a tendency for the spread of the disease under study. For his part, Hethcote [28] established the basis for the estimation of the contact number *σ*, which is the fraction of the population that comes into effective contact with an infectious individual during its infectious period. An SIR model is proposed with the categories of susceptible, infected, and recovered. In the modeling, the total population N is classified into S,I,R categories, which are homogeneously mixed and represent susceptible, infected, and recovered, respectively. In addition, consider the category of deceased individuals ℱ. It is observed that, due to effective contact, individuals move from category S to I.

Among compartmental models, the SIR model was initially selected to represent the dynamics of COVID-19 transmission and spread. This model provides a good approximation of early-stage dynamics, as it requires parameters that are relatively straightforward to define and estimate from readily available epidemiological data. Notably, the SIR model enables the calculation of the disease’s basic reproductive number, offering valuable criteria for decision-making by government and public health authorities in managing the outbreak.

Furthermore, this model can be easily extended to accommodate the possibility of reinfection, whether due to waning immunity to previously encountered strains or exposure to new variants.

Let *α*,*β*,*γ* be the rates of effective contacts between susceptible and infected, recovery of infected and mortality of infected due to the disease under study.

Under the deterministic view, the system of Eqs 1 and 2 in autonomous differences is considered, with the state variables and parameters already described.


$$S_{t}=S_{t-1}-\frac{\alpha }{{\left(S+I+R\right)}_{t-1}}S_{t-1}I_{t-1}\;;\;I_{t}=I_{t-1}+\frac{\alpha }{{\left(S+I+R\right)}_{t-1}}S_{t-1}I_{t-1}-\beta I_{t-1}-\gamma I_{t-1}$$ (1)

$$R_{t}=R_{t-1}+\beta I_{t-1}\;;\;F_{t}=F_{t-1}+\gamma I_{t-1}$$ (2)

As $\frac{dN}{dt}\neq 0$, to preserve the mass action principle, the effective contact rate is considered, normalized by the size of the epidemic survivor population. Considering *N*_*ε*_: virus-surviving population, the susceptible population in the non-trivial equilibrium is: $S^{*}=\frac{\beta +\gamma }{\alpha }N_{\varepsilon }$. Therefore, if $N_{\varepsilon }>S^{*}$, the epidemic tends to grow, leading to the basic reproductive number of the epidemic, which is $R_{0}=\frac{\alpha }{\beta +\gamma }$, representing the number of secondary cases generated by an infected individual. Only when ℛ_0_<1, the epidemic be on a path to extinction.

### Deterministic exploration

To generate some simulation scenarios of the epidemic in the city of Santiago de Cali, the parameter *α* is the average number of risky interactions $n_{r}$ per day times the probability of becoming infected $p_{c}$ in an interaction, i.e., $\alpha =n_{r}p_{c}$. In addition, *β* is the daily recovery rate, that is, the rate of recovery $p_{r}$ divided by the average duration of infection in an individual, $\beta =p_{r}/t_{i}$. Finally, *γ* is the daily mortality rate, i.e., the mortality rate $p_{m}$ among the number of days the infection lasts in an individual, $\gamma =p_{m}/t_{i}$. In addition, *β*+*γ* is the sum of the competing risks of an individual leaving the infected state and becoming recovered or deceased. Consequently, using the Poisson process, the relationship between ${\left(\beta +\gamma \right)}^{-1}=t_{i}$ is established, where $t_{i}$ is the time that, on average, an infected individual socializes by spreading the disease, either symptomatically or asymptomatically. This relationship is expressed as $\beta =\frac{1}{t_{i}}-\gamma$. To better illustrate these concepts, some simulation scenarios are presented in S2 Table.

The city of Santiago de Cali is located at coordinates 3°27′00″N 76°32′00″W, in the Valle del Cauca region, at an altitude of 1000 masl and has a population of 2.297.230 inhabitants. The city’s Gini Index is 0.52 and it has a tropical savanna climate, with an average rainfall of 1483 mm and an average temperature of 24°C [29]. Given this complex combination of factors, it is difficult to aspire to achieve optimal epidemiological parameters.

The scenario chosen for the model corresponds to the period between 13/03/2020 and 15/11/2020. This scenario is reflected in the set of parameters shown in Table 2.

**Table 2:** Basic reproductive number estimation scenario (*R*_0_).

$p_{r}$ [0.95; 0.97]	$p_{m}$ [0.03; 0.5]	$p_{c}$ [0.15; 0.2]	$t_{i}$ [7.14]	$n_{r}$ [2.3]	$\beta =p_{r}/t_{i}$	$\gamma =p_{m}/t_{i}$	$\alpha =n_{r}p_{c}$	$R_{0}=\frac{\alpha }{\beta +\gamma }$
0.95	0.05	0.1	10	2.3	9.500E-02	5.000E-03	2.30E-01	2.4

During the mathematical modeling period, a very high proportion of the population in public spaces in Santiago de Cali showed that they did not wear masks and did not respect the social distancing norms recommended by the public health authorities. In this regard, the Ministry of Health and Social Protection recommended intensifying information campaigns in the districts where the population with the highest monetary poverty rate lives.

**Fig 2:**
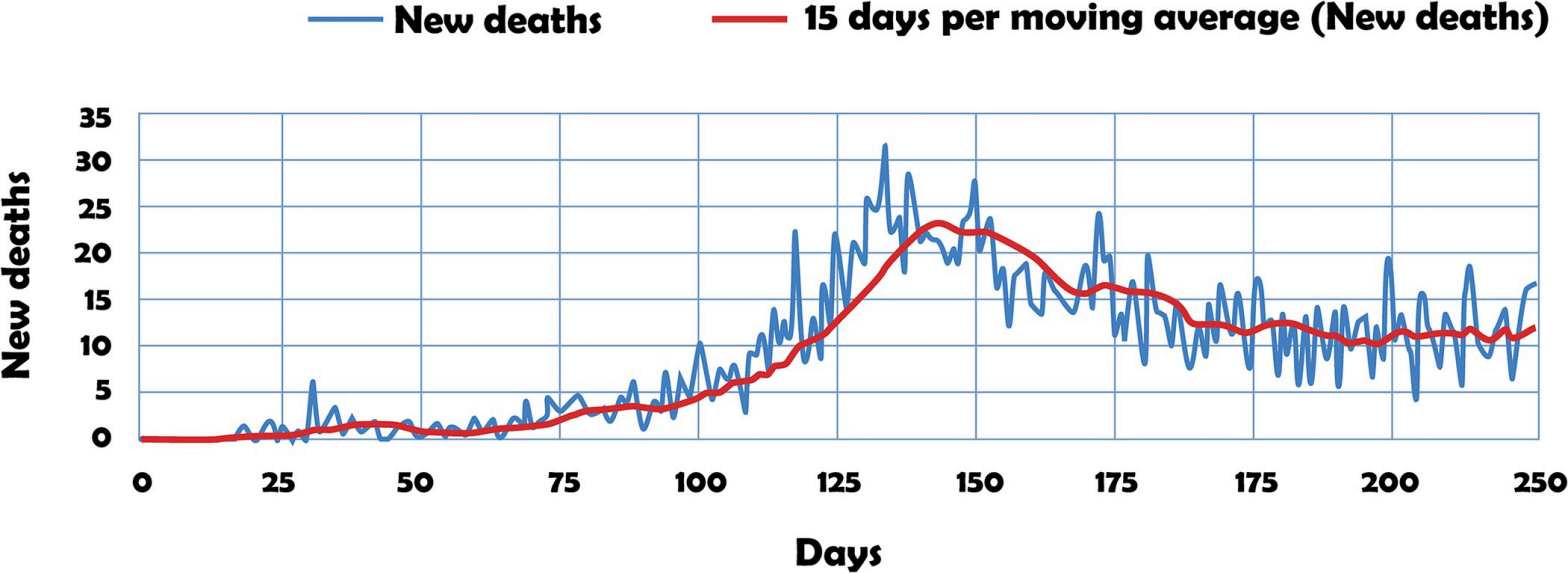
First wave of deaths in Santiago de Cali city. *R*_0_ = 2.4.

Fig 2 shows the dynamics of new deaths under simulation in the period 13/03/2020–15/11/2020.

In the scenario in which *R*_0_ = 2.4, Fig 2 represents the first wave of deaths by mathematical modeling. For this purpose, the difference equation $F_{t}=F_{t-1}+\gamma I_{t-1}$ is implemented, in which $\gamma I_{t-1}$ represents the new cases at time t.

**Fig 3:**
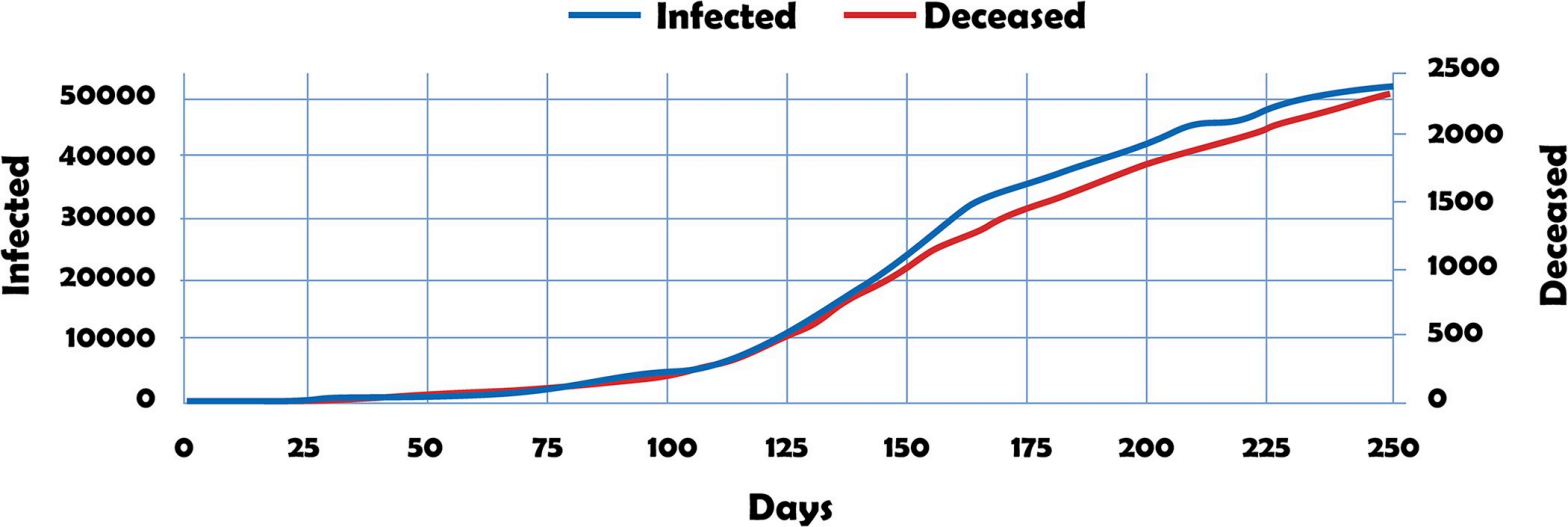
Comparative epidemiological dynamics in Santiago de Cali city. Cumulative deaths.

Fig 3 shows the dynamics of cumulative infected and deceased under simulation in the period 13/03/2020–15/11/2020.

The spread of COVID-19 in Santiago de Cali was subject to constant changes due to government-implemented control measures and public health entities. To validate the predictive value of the proposed model and provide more detail, an analysis was conducted using data spanning 246 days, divided into two datasets. The first dataset was used to develop the model, and the second to validate its predictions. S1 Fig shows actual pandemic data, while S2 Fig depicts simulations generated by the deterministic model of difference equations representing COVID-19 dynamics in Santiago de Cali.

The statistical section details the real data generated by the NIH [7], encompassing new and cumulative cases of infections, recoveries, and deaths, from March 13 to November 15, 2020.

### Inertia of cumulative deaths

According to the PHS data, the dispersion of the time series of cumulative deaths in Santiago de Cali, for the period 13/03/2020 to 30/06/2022 suggests that the relationship is not linear. A sigmoidal behavior is observed. To adjust the dispersion to a sigmoidal curve, the Gompertz curve was chosen because it adapts well to the epidemiological characteristics of the mortality rhythm, initially slow, with subsequent acceleration and final deceleration with equilibrium [30].

The Gompertz model is a flexible tool that can be fitted to real data. These characteristics make it a useful tool for research and decision making in public health. This mathematical model was proposed by Benjamin Gompertz in 1825 to explain the behavior of human deaths [26]. The Gompertz equation is presented as $F=\alpha e^{\left(-e^{\left(\beta -\varphi t\right)}\right)}$, where *α*,*β*,*φ* are constants that have independent or combined meanings in the given context. The fundamental quantitative features of the model are presented below.

Since the limit of ℱ when t grows without boundary is ℱ* = *α*, then the cumulative deceased function tends to a stationary level *α*. Eq 3 represents the second derivative of the deceased function.


$$\frac{d^{2}F}{dt^{2}}=\alpha \varphi e^{-e^{\left(\beta -\varphi t\right)}}e^{\left(\beta -\varphi t\right)}(\varphi e^{\left(\beta -\varphi t\right)}-\varphi )$$ (3)

**Fig 4:**
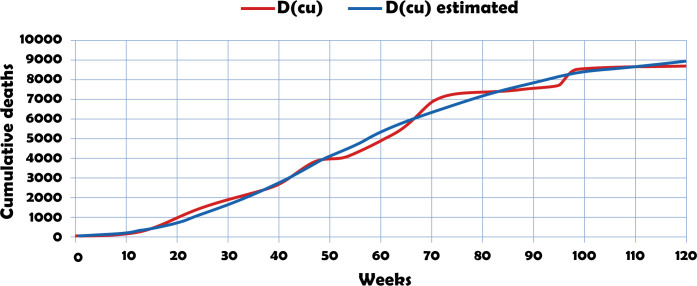
Comparative epidemiological dynamics in Santiago de Cali city. Cumulative deaths.

Equating it to zero gives *e*^(*β*−*φt*)^ = 1, from which it follows that *t* = *β*/*φ* with respective value ℱ = *α*/*e*. The inflection point of the Gompertz curve is *p*(*β*/*φ*,*α*/*e*). Moreover, the maximum growth rate of ℱ occurs at $\frac{dF}{dt}(\frac{\beta }{\varphi })=\alpha \varphi e^{-e^{\left(\beta -\varphi t\right)}}e^{\left(\beta -\varphi t\right)}$, which is equivalent to saying that ${\frac{dF}{dt}}_{max}=\alpha \varphi /e$. Fig 4 describes the dynamics of observed and estimated cumulative fatalities under simulation in Santiago de Cali in the period 13/03/2020–30/06/2022.

The goodness of fit of the Gompertz model with respect to the observed data is assessed by Pearson’s correlation coefficient, expressed as the coefficient of determination $R^{2}=1-\frac{\sum {\left(Y_{i}-{\hat{Y}}_{i}\right)}^{2}}{\sum {\left(Y_{i}-{\bar{Y}}_{i}\right)}^{2}}$, where a value of *R*^2^ close to 1 in the Gompertz model signifies a satisfactory fit of the point cloud to the curve generated by the nonlinear model.

Authors such as Hao et al. [31], found a value of *R*^2^ = 0.9997 for Wuhan, China in the first 80 days of the pandemic. Locally, if one considers that ℱ_*i*_ represents the observed deaths at time *i*, that ${\hat{F}}_{i}$ is the simulation-estimated deaths at time *i*, and that ${\bar{F}}_{i}$ is the average deaths observed at time *i*, then the correlation coefficient is defined in Eq 4.


$$R^{2}=1-\frac{\sum {\left(F_{i}-{\hat{F}}_{i}\right)}^{2}}{\sum {\left(F_{i}-{\bar{F}}_{i}\right)}^{2}}$$ (4)

Model fitting involves using the Gauss-Newton numerical method for nonlinear least squares [32], with a 95% confidence level at all intervals, up to 50 iterations and a tolerance of 10–5. This method performs successive iterations to find the most appropriate values for the model parameters from an initial set of parameters. These optimal values are those that minimize the sum of the squares of the errors, which represent the differences between the observed values of the variable under study and the values estimated by modeling.

The analysis of the series of cumulative deaths in Santiago de Cali using the Gompertz curve results in the curvature function represented by Eq 5.


$$K(t)=\frac{\left|F^{\prime \prime }(t)\right|}{{\left[1+{\left(F^{\prime }(t)\right)}^{2}\right]}^{\frac{3}{2}}}=\frac{\left|\alpha \varphi e^{\left(-e^{\left(\beta -\varphi t\right)}\right)}e^{\left(\beta -\varphi t\right)}(\varphi e^{\left(\beta -\varphi t\right)}-\varphi )\right|}{{\left[1+{\left(\alpha \varphi e^{\left(-e^{\left(\beta -\varphi t\right)}\right)}e^{\left(\beta -\varphi t\right)}\right)}^{2}\right]}^{3/2}}$$ (5)

The curvature function plays an important role in mathematical epidemiology by analyzing the long-term trend of the variable of interest. In the case of the cumulative fatality series, the curvature does not change significantly in the short term, representing increases in the level with slight differences in the rate. In the long run, the cumulative fatality curve will reach its maximum curvature point and quickly begin to decrease toward the asymptotically stable state.

## Results and discussions

According to Fig 1, since the beginning of the pandemic, an acceleration in the number of cases has been observed, while between weeks 70 and 93, an average tendency to deceleration has been observed. In other words, the growth rates of the curves are increasingly higher in the first stage and lower in the second. The recovered and deceased curves grow almost in line with the trend in the growth of the infected level.

The PHS figures confirm that, despite quarantine, social distancing, and biosecurity measures, during the first 70 weeks of the pandemic in Santiago de Cali, both the levels and rates of growth in the incidence of infected, recovered, and deceased were alarming at many points. During this period, there were three peaks that represented challenges for the human talent of the city’s health services.

In the first 70 weeks, the average rate of increase in deaths was 98 per week, while for the next 50 weeks the average rate was 37 deaths per week. However, the highest peak of the 120 weeks under study was observed in the last period.

Table 1 presents data related to the category of deaths, including the duration of each wave, the mean and the standard deviation. To determine the statistical significance of the third wave, a normal distribution is considered, where P(μ−σ<x<μ+σ)=1−2P(Z>1)=1−2*0.1587=0.6826, with $Z=\frac{X-\mu }{\sigma }$ being the normal typed variable. The third wave stands out as being the longest, lasting 28 weeks and averaging 118 deaths per week. With a probability of about 70%, the death data in this third wave are distributed around the highest average value, ranging from 40 to 196 deaths per week. In addition, the third wave shows the largest difference in the number of deaths compared to the previous wave.

The 28-week duration of the third epidemiological wave can be considered a warning period rather than a hotspot. This period is between weeks 53 and 80 of the pandemic, which corresponds to the period 20/03/2021 to 25/09/2021. During this period, between April 28, 2021 and July 20, 2021, the city of Santiago de Cali experienced a social outbreak characterized by strikes, violent protests, social unrest and the destruction of public property [33].

Between weeks 60 and 62, the figures in the epidemiological category of deaths showed a decreasing trend; however, this trend was reversed and prolonged, generating the most devastating period of the pandemic in Santiago de Cali. These weeks coincide with the incubation period of the virus in the organisms of the large crowds that participated in the social protests. For the most part, these conglomerations did not follow the recommendations provided by public health authorities to protect themselves against Covid-19 infection.

The set of parameters in Table 2 results in a moderate basic reproductive number, *R*_0_ = 2.4, which is associated with an average infection spread time of ten days. This indicates that the infected detection campaigns may not have been implemented very early.

If the average number of risky interactions (high risk of infection) for the total population of Cali was *n*_*r*_ = 2.3, then for the highest risk group it was between eight and ten per day. This means that the tendency to disregard the recommendations of social isolation on the part of a segment of the population meant that, on average, the city in the study period was projecting itself towards a collapse of the health system.

This, together with an average recovery and mortality rate, infection propagation time and probability of infection in a moderate risky interaction, represents a set of conditions that generate, under mathematical modeling, a high expectation of lethality of the virus. The modeling carried out according to the conditions reflected in the exposed parameters, indicates that Santiago de Cali would have a peak of infected people approximately in the 20th week after the beginning of the wave of infections, that is, between July and August 2020, as confirmed at the time by the series of observed data from the NIH.

According to Fig 2, explorations conducted in the field of deterministic mathematical epidemiology have generated scientific evidence indicating that a vaccine or effective treatment could significantly increase the recovery rate *β*. This, in turn, would reduce the value of *R*_0_ to values less than one, marking the beginning of the path towards disease extinction.

Information obtained through simulations and that directly observed in the course of disease surveillance generally do not coincide exactly. However, the differentials observed in Fig 3 can be minimized by improving processes such as epidemiological surveillance, updating statistical databases, and intensifying sample collection and processing, among others.

Modeling in quantitative epidemiology provides results that bring scientific accuracy to the parameters to be controlled and the direction in which such control should be exercised. This paves the way for the generation of precise and effective public policies.

Locally, considering Eq 4, a correlation coefficient *R*^2^ = 0.9939, is generated, which is scientific evidence of a high fit between the observed and estimated curve.

Table 3 summarizes the correlational adjustments and derived estimates over time.

**Table 3:** Combined report of the non-linear correlation adjustment and derived estimates.

Gompertz Model	$F=\alpha e^{\left(-e^{\left(\beta -\varphi t\right)}\right)}$		
Fitting Algorithm	Gauss-Newton		
Max iterations	200		
Tolerance	0.00001		
Initial values of the para meters	*α*_*o*_ = 8711	*β*_*o*_ = 1	*φ*_*o*_ = 1
Estimated para meters	*α* = 9557.99	*β* = 1.65	*φ* = 0.04
Estimated ES	107.63	0.036	0.001
95% CI	(9357.99;9773.08)	(1.59;1.73)	(0.03;0.04)
Iterations performed	12		
SSE Final	7157477		
DFE	117		
MSE	61175.0		
S	247.34		
Coefficient of determination *R*^2^	0.9939		
Derived estimates			
End of explosive stage *t* = *β*/*φ*	41.25		
Maximum growth rate ${\frac{dF}{dt}}_{max}=\alpha \varphi /e$	140.67		
Observed distribution for residuals	Normal		

The analysis of the curvature of the trend of the cumulative death series, calibrated with the Gompertz curve, reveals an absolute maximum value at t = 198.56 for t ≥ 0. In practical terms, this indicates that week 199 will witness a change in the trend toward asymptotically stable equilibrium in the cumulative deaths curve. The long-term epidemiological evolution for cumulative deaths is characterized by a linear trend between weeks 1 and 195, followed by a change in the trend towards equilibrium in week 196. The maximum break, with a degree of curvature K = 0.01536, occurs in week 199, with a cumulative level of 9.540 deaths.

The inertial dimension of the trend of cumulative deaths in Santiago de Cali allows a forecast to be made in a short period of time that generates warning signals to health authorities in the sense that the contingency has not yet ended, and epidemiological surveillance should be maintained to avoid new outbreaks of the virus. Table 4 shows the forecast of accumulated deaths up to the year 2023.

**Table 4:** Projected cumulative deaths extended to the end of the third year of the pandemic.

Week	Cumulative deaths estimated	Week	Cumulative deaths estimated
121	8958	139	9241
122	8979	140	9252
123	8999	141	9263
124	9018	142	9273
125	9037	143	9283
126	9055	144	9293
127	9073	145	9302
128	9090	146	9311
129	9106	147	9320
130	9122	148	9328
131	9137	149	9336
132	9151	150	9344
133	9166	151	9352
134	9179	152	9359
135	9193	153	9366
136	9205	154	9373
137	9218	155	9379
138	9230	156	9386

The S1 and S2 Figs present graphs depicting the evolution of the growth of the cumulative number of infections, recoveries, deaths, and daily new cases over a 246-day period. These graphs were generated using both real data and simulated data based on the proposed model.

S1 Fig shows the real data, while S2 Fig shows the simulated data. A strong correlation between the increasing or decreasing trends of both datasets is evident.

It is important to note that the data generated from a mathematical simulation model may, at times or over certain periods, align in trends but differ in levels when compared to the statistics reported by public health authorities. The discrepancy between the two data sets may narrow as sample collection, epidemiological tracking of positive cases, and timely and consistent data systematization intensify.

A feedback loop exists between public health data and mathematical model results, as the statistical significance of model parameters depends on the quality and quantity of the input data. However, the primary goal of epidemiological modeling is to identify critical control parameters and in what direction that control should be exerted.

## Conclusions

This study used a biomathematical model based on difference equations to analyze the transmission of Covid-19 in Santiago de Cali from March 2020 to June 2022. The results highlight the possibility of infections in the population, but effective control can limit symptomatic cases. In addition, the cumulative fatality curve has been fitted to the Gompertz model, using the Gauss-Newton method for parameter estimation.

The scientific community faces the challenge of understanding community interactions and the spread of the virus accurately. Government authorities, for their part, must make crucial decisions about limited resources and educational and control measures to maintain health care capacity in the face of symptomatic cases and prevent spillover.

Control decisions, especially in limiting population mobility, must be aligned with the dynamics suggested by mathematical models [34]. Otherwise, one would inexorably be in scenarios of chaotic trends that could generate catastrophic results.

NIH statistics and scientific research should converge in governmental management models with informed public policies, avoiding improvisation. Decision-making backed by sound data and analysis is essential to address challenges effectively and strategically.

The basic epidemic reproductive number is $R_{0}=\frac{\alpha }{\beta +\gamma }$, decreasing with decreasing contact rate *α* and increasing recovery and mortality rates, *β*,*γ* respectively. It is paradoxical that an increase in mortality decreases the probability of effective contact between individuals. In practice, control by increasing mortality is unfeasible, focusing on increasing recovery and reducing effective contacts.

This study highlights that the variations in the dynamics of infected, recovered and deceased, in short periods, may be influenced by non-structural aspects such as changes in sampling and efficient delivery of results. However, in the long term, it is evident that rigorous epidemiological follow-up, attention to health personnel and public policies based on scientific consensus are fundamental for modeling trajectories. These results underscore the importance of collaboration between the scientific community and public health officials for effective epidemic management.

For future work, a time series study is needed to analyze the rates of susceptible, infected, recovered, and deceased persons by age in Santiago de Cali. This to understand the impact of the social outbreak on the spread of Covid-19, given that different age groups face different risks. In addition, a geospatial study by communes or neighborhoods is proposed, since the spread of Covid-19 may vary according to demographic, social and geographic factors. The results would guide the allocation of resources for the containment of Covid-19 and future viruses at the regional level.

## Supporting information

S1 Table Covid-19: New cases in Santiago de Cali city (13/03/2020–30/06/2022). (PDF)

S2 Table Basic and derived parameters for some simulation scenarios of the Covid-19 epidemic in Santiago de Cali city. (PDF)

S3 Table Covid-19: New cases in Santiago de Cali city. Simulation-generated data.
(PDF)

S1 Fig Covid-19/real data for Santiago de Cali, time in days from 13/3/2020 to 15/11/2020. (a) Epidemiological situation Infected, Removed, F (deaths); (b) Accumulated deaths; (c) New deaths.
(PDF)

S2 Fig Covid-19: Simulated data for Santiago de Cali, time in days (1 to 246). (a) Epidemiological curves Infected, Removed, F (deaths); (b) Accumulated deaths; (c) New deaths.
(PDF)
