# Supplementary material for: Biomathematical model to analyze the transmission dynamics of Covid-19: Case study, Santiago de Cali, Colombia
Source: PLoS One. 2024 Dec 2;19(12):e0311414. doi: 10.1371/journal.pone.0311414 (PMC11611158; doi:10.1371/journal.pone.0311414)
Supplement: S1 Table — (PDF) [file pone.0311414.s001.pdf]

S1 Table. Covid-19: new cases in Santiago de Cali City (13/03/2020 - 30/06/2022).

Week	I	R	F	N/A	Week	I	R	F	N/A	Week	I	R	F	N/A
**1**	15	14	0	0	**41**	5276	5152	111	13	**81**	940	918	15	7
**2**	41	39	2	0	**42**	4762	4595	147	20	**82**	771	756	11	4
**3**	74	67	7	0	**43**	3809	3638	147	24	**83**	860	824	29	7
**4**	243	218	25	0	**44**	5115	4909	179	27	**84**	726	707	17	2
**5**	98	92	6	0	**45**	5042	4858	146	38	**85**	817	798	12	7
**6**	160	152	8	0	**46**	8217	7943	219	55	**86**	701	673	23	5
**7**	200	187	12	1	**47**	8926	8727	150	49	**87**	917	895	18	4
**8**	219	204	13	2	**48**	3692	3567	95	30	**88**	974	939	29	6
**9**	280	261	19	0	**49**	2848	2775	55	18	**89**	1060	1036	20	4
**10**	483	452	28	3	**50**	2135	2074	50	11	**90**	1136	1104	28	4
**11**	731	702	25	4	**51**	1625	1571	45	9	**91**	1137	1103	31	3
**12**	675	637	36	2	**52**	1661	1607	44	10	**92**	1150	1121	21	8
**13**	915	878	32	5	**53**	2063	2010	44	9	**93**	2086	2050	29	7
**14**	1183	1121	56	6	**54**	2375	2305	58	12	**94**	9140	9088	34	18
**15**	1577	1506	60	11	**55**	2836	2757	68	11	**95**	25284	25154	82	48
**16**	1830	1741	69	20	**56**	4130	4017	98	15	**96**	24239	23838	248	153
**17**	1967	1858	97	12	**57**	5471	5279	156	36	**97**	14600	14103	302	195
**18**	2412	2305	95	12	**58**	4992	4834	129	29	**98**	7090	6797	158	135
**19**	2858	2707	137	14	**59**	4384	4219	144	21	**99**	3516	3390	64	62
**20**	3865	3685	160	20	**60**	3903	3748	104	51	**100**	1723	1646	35	42
**21**	4256	4078	158	20	**61**	5189	5003	147	39	**101**	1576	1546	15	15
**22**	5032	4873	128	31	**62**	3248	3101	127	20	**102**	848	807	24	17
**23**	4447	4267	144	36	**63**	5121	4951	148	22	**103**	572	558	5	9
**24**	2657	2549	92	16	**64**	6101	5909	163	29	**104**	274	256	5	13
**25**	2666	2574	69	23	**65**	7607	7361	219	27	**105**	196	187	3	6
**26**	2510	2412	80	18	**66**	6725	6519	182	24	**106**	233	216	12	5
**27**	2846	2768	57	21	**67**	11809	11520	252	37	**107**	191	179	7	5
**28**	2590	2492	74	24	**68**	11018	10733	248	37	**108**	147	144	1	2
**29**	2703	2604	77	22	**69**	10349	10035	274	40	**109**	126	123	2	1
**30**	3141	3060	59	22	**70**	10098	9839	227	32	**110**	75	75	0	0
**31**	2834	2742	71	21	**71**	6595	6432	138	25	**111**	69	67	1	1
**32**	2923	2823	85	15	**72**	5668	5539	106	23	**112**	39	39	0	0
**33**	3866	3778	65	23	**73**	4333	4246	66	21	**113**	73	71	1	1
**34**	3805	3711	73	21	**74**	3297	3227	57	13	**114**	120	120	0	0
**35**	3877	3782	84	11	**75**	3867	3779	54	34	**115**	251	251	0	0
**36**	4002	3892	100	10	**76**	1627	1580	30	17	**116**	302	296	1	5
**37**	3238	3135	95	8	**77**	978	951	22	5	**117**	414	409	2	3
**38**	3920	3798	103	19	**78**	1007	988	13	6	**118**	906	896	4	6
**39**	4022	3905	98	19	**79**	1165	1152	11	2	**119**	1618	1595	7	16
**40**	4235	4124	98	13	**80**	803	789	12	2	**120**	2848	2786	33	29

Not available (N/A)
