# Supplementary material for: Biomathematical model to analyze the transmission dynamics of Covid-19: Case study, Santiago de Cali, Colombia
Source: PLoS One. 2024 Dec 2;19(12):e0311414. doi: 10.1371/journal.pone.0311414 (PMC11611158; doi:10.1371/journal.pone.0311414)
Supplement: S2 Table — (PDF) [file pone.0311414.s002.pdf]

S2 Table. Basic and derived parameters for some simulation scenarios of the Covid-19 epidemic in Santiago de Cali city.

**Scen.**	p_r $[0.95, 0.97]$	p_m $[0.03, 0.05]$	p_c $[0.15, 0.2]$	t_i $[7, 14]$	n_r $[2, 3]$	$\beta = p_r/t_i$	$\gamma = p_m/t_i$	$\alpha = n_r p_c$	$\mathcal{R}_0 = \frac{\alpha}{\beta+\gamma}$
**1**	0.96	0.04	0.175	10.5	2.0	9.143E-02	3.810E-03	3.50E-01	3.7
**2**	0.96	0.04	0.175	10.5	3.0	9.143E-02	3.810E-03	5.25E-01	5.5
**3**	0.96	0.04	0.175	7.0	2.5	1.371E-01	5.714E-03	4.38E-01	3.1
**4**	0.96	0.04	0.175	14.0	2.5	6.857E-02	2.857E-03	4.38E-01	6.1
**5**	0.96	0.04	0.150	10.5	2.5	9.143E-02	3.810E-03	3.75E-01	3.9
**6**	0.96	0.04	0.200	10.5	2.5	9.143E-02	3.810E-03	5.00E-01	5.3
**7**	0.97	0.03	0.175	10.5	2.5	9.238E-02	2.857E-03	4.38E-01	4.6
**8**	0.95	0.05	0.175	10.5	2.5	9.048E-02	4.762E-03	4.38E-01	4.6
**9**	0.96	0.04	0.175	10.5	2.5	9.143E-02	3.810E-03	4.38E-01	4.6
**10**	0.97	0.03	0.100	7.0	2.0	1.386E-01	4.286E-03	2.00E-01	1.4
