# Supplementary material for: Biomathematical model to analyze the transmission dynamics of Covid-19: Case study, Santiago de Cali, Colombia
Source: PLoS One. 2024 Dec 2;19(12):e0311414. doi: 10.1371/journal.pone.0311414 (PMC11611158; doi:10.1371/journal.pone.0311414)
Supplement: S3 Table — Simulation-generated data. (PDF) [file pone.0311414.s003.pdf]

S3 Table. Covid-19: new cases in Santiago de Cali City. Simulation-generated data.

t	**Accumulated cases**			**New**	***t***	**Accumulated cases**			**New**	***t***	**Accumulated cases**			**New**
**Days**	I	R	F	F	**Days**	I	R	F	F	**Days**	I	R	F	F
**0**	1	0	0	0	**83**	2989	2543	113	3	**166**	33786	49842	1297	15
**1**	1	0	0	0	**84**	3066	2661	115	2	**167**	34044	51180	1310	14
**2**	1	0	0	0	**85**	3183	2781	117	3	**168**	34333	52526	1326	16
**3**	1	0	0	0	**86**	3308	2903	123	5	**169**	34675	53880	1346	20
**4**	3	0	0	0	**87**	3356	3032	126	4	**170**	34849	55252	1360	14
**5**	8	0	0	0	**88**	3521	3159	134	7	**171**	35062	56622	1385	25
**6**	8	1	0	0	**89**	3637	3297	136	3	**172**	35562	58005	1404	19
**7**	8	1	0	0	**90**	3751	3442	137	1	**173**	35776	59408	1423	19
**8**	9	1	0	0	**91**	4011	3590	139	2	**174**	36116	60828	1435	11
**9**	16	2	0	0	**92**	4085	3745	144	5	**175**	36283	62258	1449	14
**10**	20	2	0	0	**93**	4338	3906	147	3	**176**	36616	63700	1459	10
**11**	23	3	0	0	**94**	4389	4071	155	8	**177**	36901	65147	1476	17
**12**	47	4	0	0	**95**	4565	4245	157	2	**178**	36950	66608	1491	15
**13**	48	6	0	0	**96**	4696	4421	164	7	**179**	37300	68078	1499	8
**14**	50	8	0	0	**97**	4867	4602	170	6	**180**	37504	69550	1519	20
**15**	50	10	0	0	**98**	5088	4793	174	4	**181**	37920	71036	1534	15
**16**	58	12	0	0	**99**	5256	4992	179	5	**182**	38217	72539	1547	14
**17**	65	14	0	0	**100**	5422	5191	190	11	**183**	38464	74053	1561	14
**18**	72	15	1	1	**101**	5615	5399	198	8	**184**	38632	75582	1571	10
**19**	81	17	2	1	**102**	5725	5620	202	4	**185**	38786	77113	1586	15
**20**	104	20	2	0	**103**	5937	5842	209	7	**186**	39072	78655	1595	9
**21**	106	25	2	0	**104**	6241	6071	217	8	**187**	39435	80210	1603	8
**22**	122	28	3	1	**105**	6526	6315	223	6	**188**	39731	81780	1611	8
**23**	135	31	5	2	**106**	6902	6568	232	8	**189**	40028	83356	1623	13
**24**	150	36	5	0	**107**	7058	6837	238	6	**190**	40394	84948	1632	9
**25**	160	40	7	2	**108**	7184	7117	240	2	**191**	40530	86549	1647	15
**26**	174	47	7	0	**109**	7404	7395	250	10	**192**	40625	88161	1657	10
**27**	251	54	7	0	**110**	7492	7683	259	9	**193**	40924	89769	1674	17
**28**	286	63	8	1	**111**	7938	7971	270	12	**194**	41193	91393	1686	13
**29**	350	74	8	0	**112**	8239	8282	277	7	**195**	41693	93029	1698	12
**30**	397	88	8	0	**113**	8507	8597	291	14	**196**	42077	94681	1714	16
**31**	403	98	14	6	**114**	8903	8927	301	10	**197**	42299	96350	1728	14
**32**	407	113	16	1	**115**	9067	9270	314	13	**198**	42473	98035	1735	8
**33**	420	127	18	2	**116**	9267	9622	325	11	**199**	42588	99716	1753	18
**34**	435	141	21	3	**117**	9581	9970	348	23	**200**	42922	101403	1770	17
**35**	454	155	24	3	**118**	9948	10339	362	14	**201**	43149	103107	1782	13
**36**	485	172	25	1	**119**	10414	10729	370	8	**202**	43567	104820	1795	13
**37**	497	189	27	2	**120**	10888	11132	383	13	**203**	43866	106556	1802	7
**38**	506	207	29	2	**121**	11164	11559	392	9	**204**	44141	108297	1815	14
**39**	546	226	31	1	**122**	11404	11989	408	17	**205**	44368	110054	1824	9
**40**	571	247	31	1	**123**	11875	12433	420	12	**206**	44401	111817	1836	12
**41**	595	268	33	1	**124**	12526	12886	442	22	**207**	44571	113588	1841	6
**42**	630	291	34	1	**125**	12843	13369	461	19	**208**	44740	115357	1855	14
**43**	648	316	34	0	**126**	13073	13869	474	13	**209**	44984	117141	1861	6
**44**	666	342	34	0	**127**	13446	14371	495	21	**210**	45305	118926	1875	14
**45**	685	368	35	1	**128**	13730	14888	517	22	**211**	45506	120728	1885	10
**46**	691	394	36	1	**129**	13990	15418	536	19	**212**	45587	122540	1894	9
**47**	721	421	37	1	**130**	14233	15951	562	26	**213**	45656	124350	1907	13
**48**	741	448	39	2	**131**	14838	16495	587	25	**214**	45709	126170	1913	6
**49**	779	477	40	1	**132**	15733	17065	611	24	**215**	45915	127984	1927	14
**50**	822	508	40	0	**133**	16134	17662	643	32	**216**	46191	129811	1937	10
**51**	865	540	40	1	**134**	16704	18285	665	22	**217**	46401	131647	1949	12
**52**	867	575	40	0	**135**	17091	18929	690	24	**218**	46625	133489	1962	13

**53**	894	607	43	2	**136**	17427	19595	707	18	**219**	46733	135341	1975	13
**54**	922	643	43	0	**137**	18025	20263	736	29	**220**	46798	137204	1982	7
**55**	963	678	44	1	**138**	18330	20960	761	25	**221**	47113	139064	1994	12
**56**	1003	715	45	1	**139**	19158	21672	782	21	**222**	47350	140940	2003	8
**57**	1040	755	46	1	**140**	19661	22416	804	23	**223**	47758	142814	2022	20
**58**	1093	796	47	1	**141**	20357	23181	826	21	**224**	48004	144712	2035	12
**59**	1103	838	49	2	**142**	20904	23973	847	22	**225**	48202	146618	2049	14
**60**	1130	879	52	3	**143**	21199	24788	869	22	**226**	48208	148534	2061	12
**61**	1158	923	52	1	**144**	21534	25617	888	18	**227**	48289	150453	2070	9
**62**	1210	968	54	1	**145**	22283	26457	909	21	**228**	48503	152381	2074	4
**63**	1276	1013	57	3	**146**	23009	27330	928	19	**229**	48718	154305	2090	16
**64**	1316	1065	57	0	**147**	23630	28227	951	24	**230**	48875	156243	2101	11
**65**	1380	1117	57	0	**148**	24023	29148	975	24	**231**	49100	158186	2113	12
**66**	1454	1171	58	2	**149**	24610	30080	1003	28	**232**	49331	160142	2120	7
**67**	1477	1226	61	2	**150**	24735	31044	1024	21	**233**	49458	162102	2134	14
**68**	1559	1284	62	2	**151**	25286	32012	1046	22	**234**	49456	164068	2146	12
**69**	1627	1341	67	5	**152**	27178	32999	1070	24	**235**	49432	166034	2159	13
**70**	1687	1406	68	1	**153**	27779	34070	1086	16	**236**	49645	168006	2164	5
**71**	1796	1471	71	2	**154**	28271	35162	1106	19	**237**	49888	169972	2183	19
**72**	1877	1541	72	2	**155**	28835	36280	1118	12	**238**	50066	171955	2196	13
**73**	1970	1611	77	5	**156**	29288	37415	1136	18	**239**	50351	173947	2207	11
**74**	2014	1686	81	3	**157**	29444	38569	1154	17	**240**	50450	175952	2216	9
**75**	2153	1764	84	3	**158**	30174	39728	1173	19	**241**	50504	177961	2225	9
**76**	2268	1846	87	3	**159**	30799	40920	1187	14	**242**	50745	179969	2237	13
**77**	2378	1933	92	4	**160**	31276	42138	1202	14	**243**	50949	181985	2251	13
**78**	2475	2023	97	5	**161**	32042	43375	1215	13	**244**	51112	184009	2265	14
**79**	2624	2118	101	4	**162**	32516	44638	1234	19	**245**	51328	186047	2271	6
**80**	2690	2219	104	3	**163**	32833	45923	1250	16	**246**	51514	188089	2282	11
**81**	2772	2324	107	3	**164**	32949	47220	1267	17	**247**	51621	190135	2297	15
**82**	2857	2432	110	3	**165**	33364	48522	1282	16	**248**	51598	192183	2314	17
