# Supplementary material for: Biomathematical model to analyze the transmission dynamics of Covid-19: Case study, Santiago de Cali, Colombia
Source: PLoS One. 2024 Dec 2;19(12):e0311414. doi: 10.1371/journal.pone.0311414 (PMC11611158; doi:10.1371/journal.pone.0311414)
Supplement: S1 Fig — (a) Epidemiological situation Infected, Removed, F (deaths); (b) Accumulated deaths; (c) New deaths. (PDF) [file pone.0311414.s004.pdf]

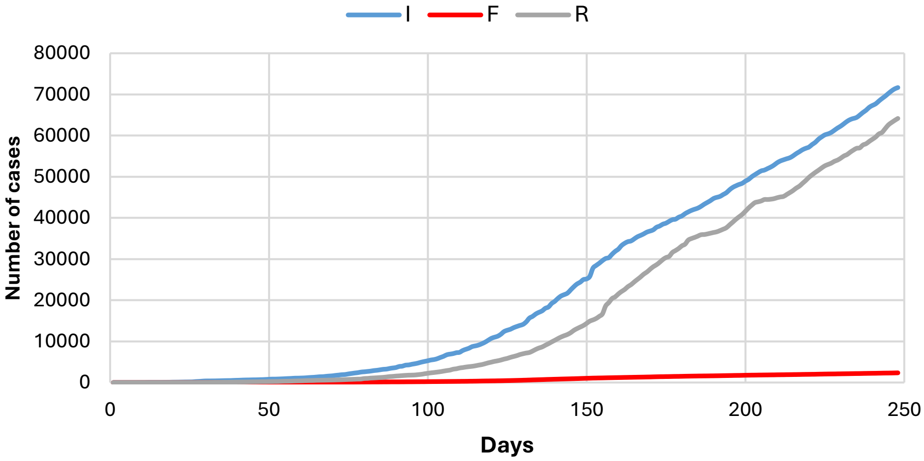

I
F
R
Number of cases
80000
70000
60000
50000
40000
30000
20000
10000
0
0
50
100
150
200
250
Days

(a)

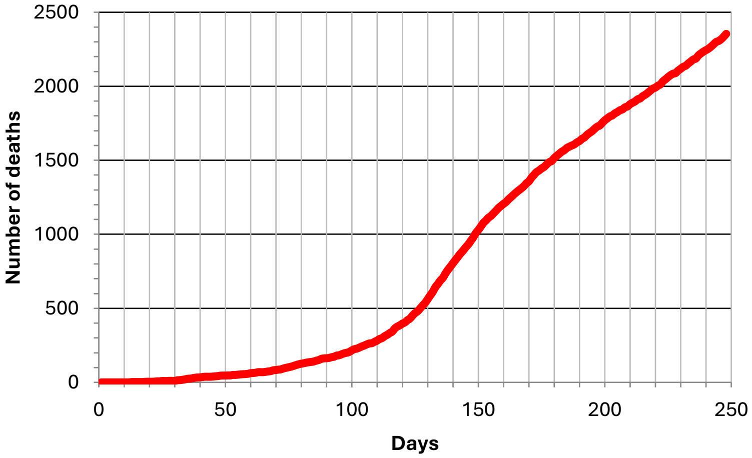

Number of deaths
2500
2000
1500
1000
500
0
0
50
100
150
200
250
Days

(b)

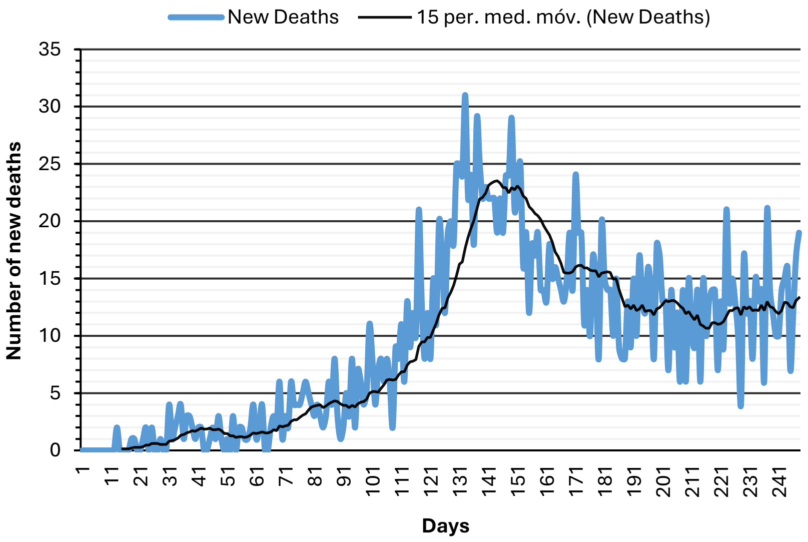

(c)

S1 Fig. Covid-19/real data for Santiago de Cali, time in days from 13/3/2020 to 15/11/2020. (a) Epidemiological situation Infected, Removed, F (deaths); (b) Accumulated deaths; (c) New deaths.
