# Supplementary material for: Biomathematical model to analyze the transmission dynamics of Covid-19: Case study, Santiago de Cali, Colombia
Source: PLoS One. 2024 Dec 2;19(12):e0311414. doi: 10.1371/journal.pone.0311414 (PMC11611158; doi:10.1371/journal.pone.0311414)
Supplement: S2 Fig — (a) Epidemiological curves Infected, Removed, F (deaths); (b) Accumulated deaths; (c) New deaths. (PDF) [file pone.0311414.s005.pdf]

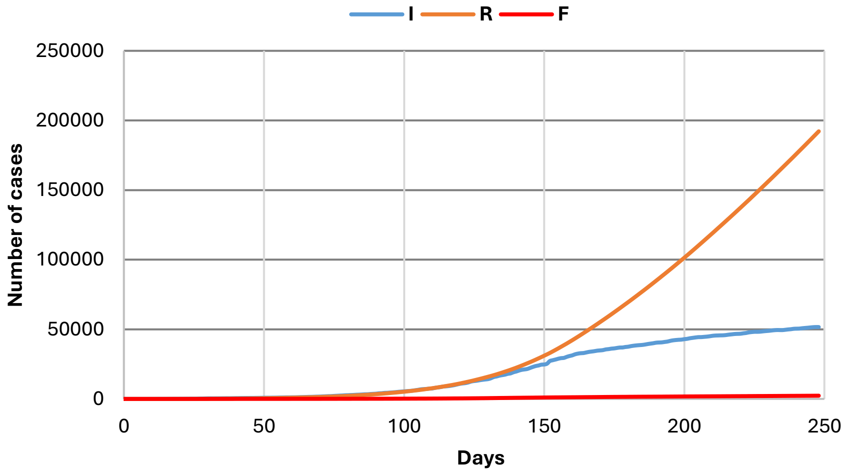
I
R
F
250000
200000
150000
100000
50000
0
Number of cases
0
50
100
150
200
250
Days

(a)

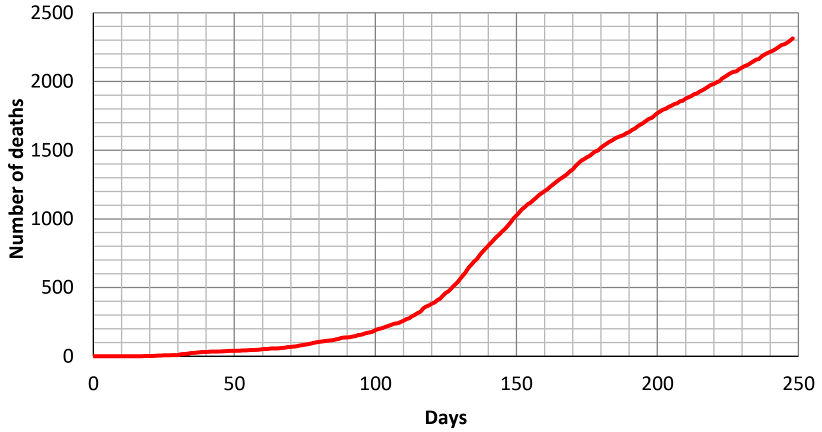
2500
2000
1500
1000
500
0
Number of deaths
0
50
100
150
200
250
Days

(b)

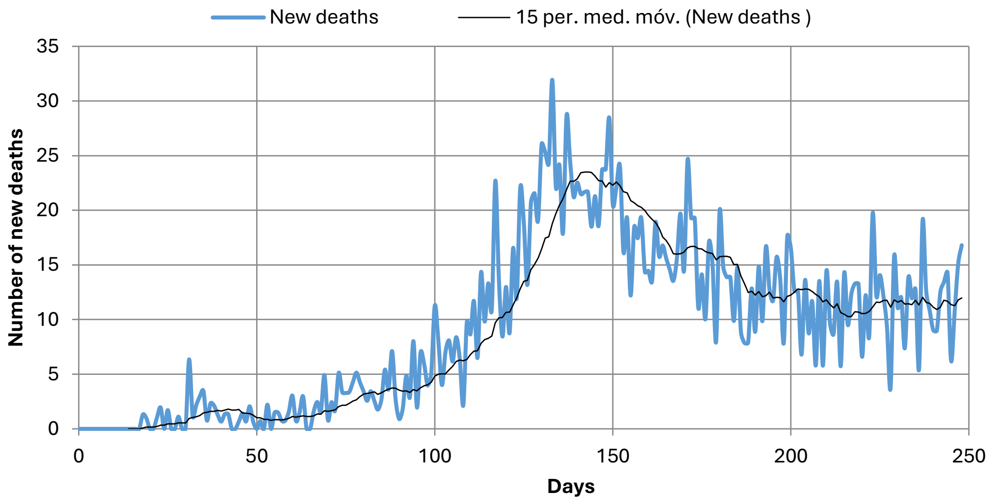

(c)

S2 Fig. Covid-19: Simulated data for Santiago de Cali, time in days (1 to 246). (a) Epidemiological curves Infected, Removed, F (deaths); (b) Accumulated deaths; (c) New deaths.
